# Supplementary material for: Effect of caffeine and other xanthines on liver sinusoidal endothelial cell ultrastructure
Source: Sci Rep. 2023 Aug 17;13:13390. doi: 10.1038/s41598-023-40227-0 (PMC10435486; doi:10.1038/s41598-023-40227-0)
Supplement: Supplementary file 1 — Supplementary Figures. [file 41598_2023_40227_MOESM1_ESM.docx]

**Supplementary information**


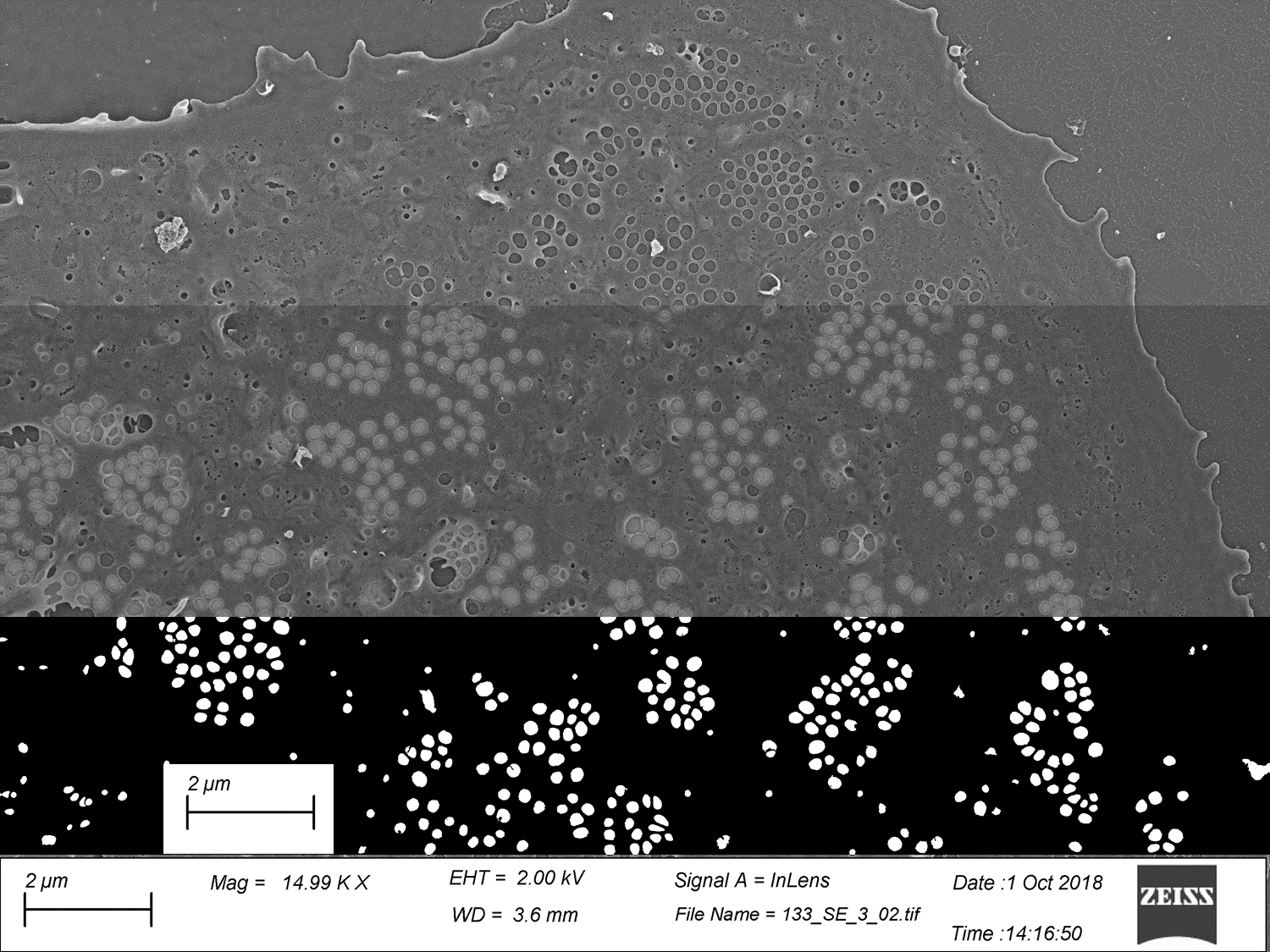
S1. Image analysis data

**Figure S1.** Examples of the images used for measurement of fenestration diameters. The top section is a raw SEM (left) / SIM (right) image, bottom section presents the binary mask used for quantitative analysis, and the middle section shows the overlay used to identify fenestrations.


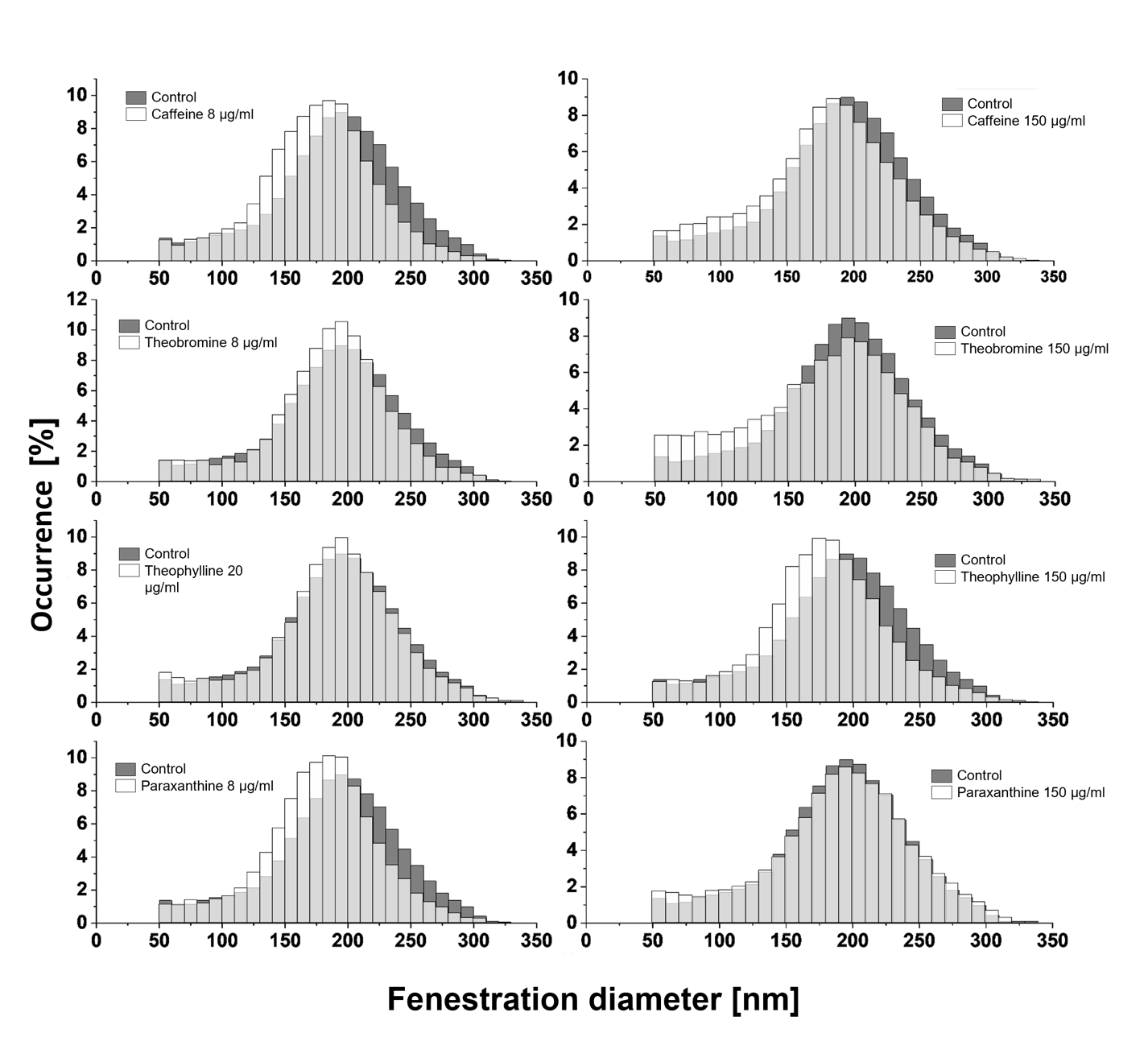
S2. Fenestration size distributions

**Figure S2.** Distribution of fenestration size after treatment, in comparison with control/untreated samples
